# To the Editor of the Medical and Physical Journal

**Published:** 1822-04

**Authors:** John Webster

**Affiliations:** 3, Little George-Street, Westminster


THE LONDON

### Medical and Physical Journal.

4 OF VOL. XLVII.] APRIL, 1822. [No. 278.

For many fortunate discoveries in medicine, and for the detection of numerous errors, the world is indebted to the rapid circulation of Monthly Journals; and there never existed any work, to which the Faculty, in Europe and America, were under deeper obligations, than to the Medical and Physical Journal of London, now forming a long, but an invaluable, series.—RUSH.

### Original Communications, Select Observations, etc.

### NOSOLOGY.

*To the Editor of the Medical and Physical Journal.*

SIR,

THE accompanying specimen of a new system of Nosology, I have just received from a very intelligent medical friend in India, Dr. ADAM, assistant physician to the General Hospital at Calcutta. As it is interesting, from its novelty,—from the peculiar views which it contains,—and from the place where it is published, I think it may prove acceptable to the profession in England: you will, therefore, oblige me by giving it an early insertion in your widely-circulated and very respectable Journal. I am, &c.

JOHN WEBSTER, M.D.

&c. &c.

*3, Little George-street, Westminster;*
*6th March,* 1822.

